# Chronic Low Dose Chlorine Exposure Aggravates Allergic Inflammation and Airway Hyperresponsiveness and Activates Inflammasome Pathway

**DOI:** 10.1371/journal.pone.0106861

**Published:** 2014-09-09

**Authors:** Sae-Hoon Kim, Da-Eun Park, Hyun-Seung Lee, Hye-Ryun Kang, Sang-Heon Cho

**Affiliations:** 1 Department of Internal Medicine, Seoul National University College of Medicine, Seoul, Republic of Korea; 2 Institute of Allergy and Clinical Immunology, Seoul National University Medical Research Center, Seoul, Korea; 3 Department of Internal Medicine, Seoul National University Bundang Hospital, Seongnam, Republic of Korea; French National Centre for Scientific Research, France

## Abstract

**Background:**

Epidemiologic clinical studies suggested that chronic exposure to chlorine products is associated with development of asthma and aggravation of asthmatic symptoms. However, its underlying mechanism was not clearly understood. Studies were undertaken to define the effects and mechanisms of chronic low-dose chlorine exposure in the pathogenesis of airway inflammation and airway hyperresponsiveness (AHR).

**Methods:**

Six week-old female BALB/c mice were sensitized and challenged with OVA in the presence and absence of chronic low dose chlorine exposure of naturally vaporized gas of 5% sodium hypochlorite solution. Airway inflammation and AHR were evaluated by bronchoalveolar lavage (BAL) cell recovery and non-invasive phlethysmography, respectively. Real-time qPCR, Western blot assay, and ELISA were used to evaluate the mRNA and protein expressions of cytokines and other inflammatory mediators. Human A549 and murine epithelial (A549 and MLE12) and macrophage (AMJ2-C11) cells were used to define the responses to low dose chlorine exposure in vitro.

**Results:**

Chronic low dose chlorine exposure significantly augmented airway inflammation and AHR in OVA-sensitized and challenged mice. The expression of Th2 cytokines IL-4 and IL-5 and proinflammatory cytokine IL-1β and IL-33 were significantly increased in OVA/Cl group compared with OVA group. The chlorine exposure also activates the major molecules associated with inflammasome pathway in the macrophages with increased expression of epithelial alarmins IL-33 and TSLP *in vitro*.

**Conclusion:**

Chronic low dose exposure of chlorine aggravates allergic Th2 inflammation and AHR potentially through activation of inflammasome danger signaling pathways.

## Introduction

Bronchial asthma is a chronic airway inflammatory disease characterized by reversible airway obstruction and airway hyperresponsiveness (AHR). Both genetic factors and environmental factors are involved in the pathogenesis of asthma. A variety of environmental factors including not only allergens but also viral infections, exposure to air pollution, smoking, and chemical irritants contribute to the development of asthma and trigger asthmatic symptoms [1].

Chlorines are widely used in various applications such as materials for disinfection and cleaning in home, public building, and industry due to low cost, ease use and strong effect of deodorizing, bleaching and germicide activity [2]. Sodium hypochlorite (NaOCl) solution commonly known as a disinfectant and bleaching agent can influence human respiratory tract by inhalation of chlorine gas naturally vaporized from the solution. Chloramines such as monochloramine (NH_2_Cl), dichloramine (NaHCl_2_) and trichloramine (NCl_3_), which are formed after hypochlorous acid (HOCl) encounter nitrogen organic compounds, can also affect human airway in forms of aerosol and gas [2]. Acute exposure of high dose chlorine gas (Cl_2_) damage airway mucosa through formation of reactive oxygen species (ROS) and can induce reactive airway dysfunction syndrome (RADS) enhancing airway inflammation and AHR and sometimes provoke acute respiratory distress syndrome (ARDS) in severe cases [3]–[5]. On the other hands, exposure to low dose chlorine gas can be ignored because it does not induce any acute symptoms. However, persons with chronic occupation exposure to low dose chlorine products such as swimming athletics, swimming pool workers, and cleaners are revealed to have higher prevalence of respiratory symptoms and asthma in the previous epidemiological studies [6]–[9]. In addition, there have been reports of the association between exposure to chlorine products and atopic dermatitis, allergic contact dermatitis and contact urticaria [10]–[13]. These suggest that chlorine exposure is related to the development of cutaneous allergic disease as well as respiratory disease.

Experimental studies have been performed about the development of respiratory allergic disease induced by chlorine exposure. Hypochlorous acid or chloramines increased epithelial or endothelial permeability by disruption of cellular junction and oxidative cellular injury [14], [15]. In animal study, acute exposure to high dose chlorine gas enhanced AHR and production of nitric oxide as like the results of epidemiologic studies [16]–[18]. However, the effects of chronic exposure to low dose chlorine combined with allergen have rarely been explored in the *in vivo* animal model yet. Moreover, the mechanism of asthma development or aggravation induced by low dose chlorine has not been fully elucidated yet.

In the present study, we aimed to define the effects and mechanism of chronic low dose chlorine exposure in the pathogenesis of airway inflammation and AHR using an animal model of asthma and airway epithelial cells and macrophages in culture. These studies identified that chronic low dose chlorine exposure aggravates the allergen-induced airway inflammation and AHR, similarly to high dose exposure as reported previously, and activates the major molecules associated with inflammasome pathways. These studies highlight chronic environmental exposure of chlorine as a significant risk factor for the development of allergic lung disease potentially through activation of inflammasome danger signaling pathways.

## Methods

### Sensitization and challenge procedure

**Figure 1:**
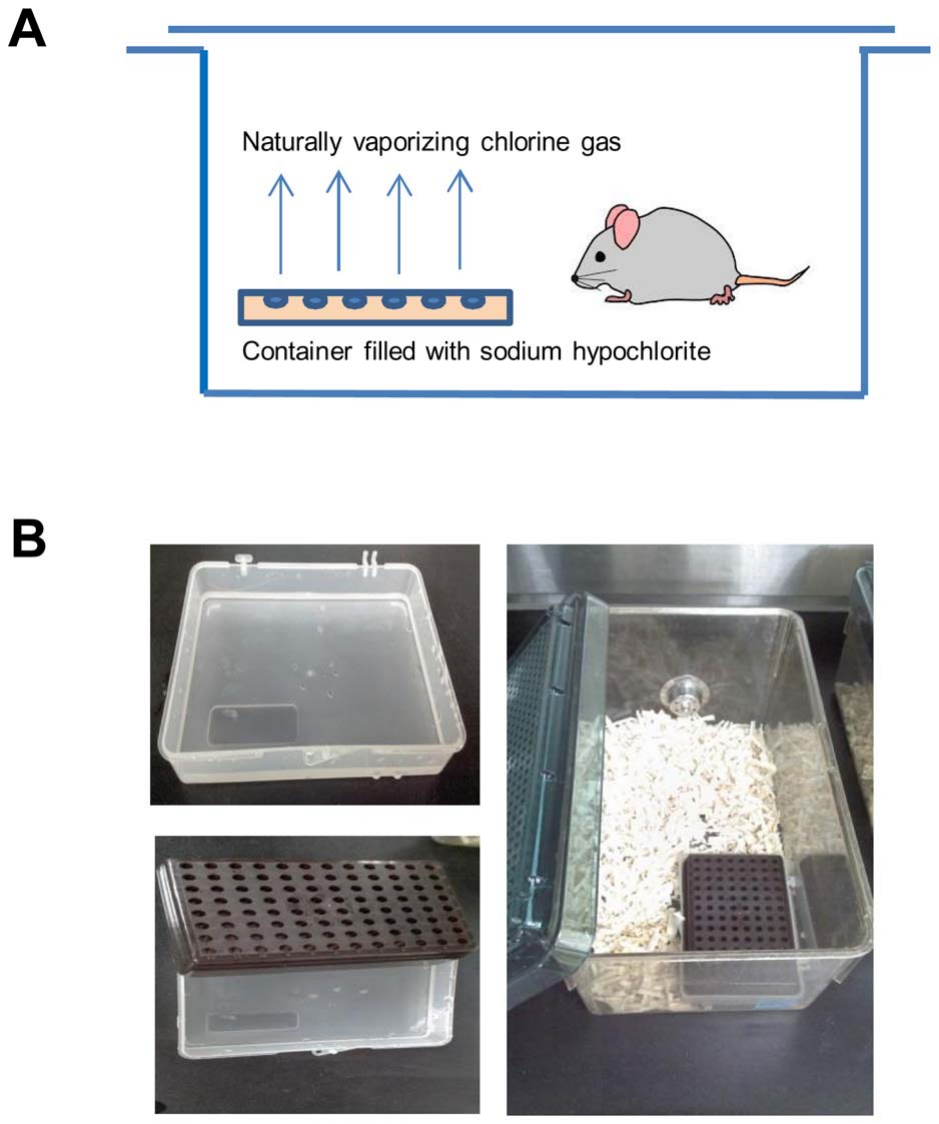
Mouse chlorine exposure system. A, a container filled with NaOCl solution was placed in the cage and mice were exposed to naturally vaporizing chlorine gas 8 hours a day, 5 times a week, for 4 weeks. B, a container with holes on the top surface to avoid direct skin contact with the NaOCl solution.

Experimental procedures were carried out with approval from Seoul National University Institutional Animal Care and Use Committee. Six-week-old, female BALB/c mice were used for the study. Mice were divided into four groups. Control group underwent neither sensitization/challenge with OVA nor 4 weeks exposure to low-dose chlorine (control; n = 6). Other groups underwent only 4 weeks exposure to low-dose chlorine (Cl group; n = 6), or only sensitization/challenge with OVA (OVA group; n = 6), or both sensitization/challenge with OVA and 4 weeks exposure to low-dose chlorine (OVA/Cl group; n = 6). Allergen sensitization was performed with 75 ug OVA (Sigma, St. Louis, MO, USA) with 2 mg aluminum hydroxide (Sigma) via intraperitoneal injection at day 1 and day 14. The mice were challenged with 50 ug OVA via intranasal route at day 28, 29, and 30 consecutively. At day 31, methacholine challenge was performed and enhanced pause (penh) was measured using one chamber plethysmography (All Medicus 2000, Anyang, Korea) for the evaluation of AHR as described previously [19]. At day 32, bronchoalveolar lavage (BAL) fluid and lung tissue were collected after sacrifice of the mice. Chlorine gas was inhaled by exposing the mice to naturally vaporizing gas from 5% sodium hypochlorite solution (NaOCl; Sigma). A container with holes on the top surface was prepared to avoid direct skin contact with the NaOCl solution. For the chronic exposure to low-dose chlorine gas, the container filled with 3 mL of 5% NaOCl solution was placed in the mouse cage 8 hours a day, 5 times a week, for 4 weeks (Fig. 1A–B). The average concentration of chlorine gas in the mouse case was 0.0001 ppm which was measured using ion chromatography with filter collection method for 8 hours,

### Lung inflammation and mucus secretion

Differential cell count was performed after Diff-quick staining of BAL fluid cells in a cytospin preparation. After collection of the blood by cardiac puncture, right lungs were removed and stored at −80°C for the measurement of cytokines and inflammatory mediators. Left lungs were inflated with 0.5% low melting point agarose gel, removed en bloc for further formalin fixation, embedded in paraffin, cut and stained with Hematoxylin and Eosin (H&E) for the detection of inflammation and Periodic-Acid-Schiff (PAS) for analysis of mucus secretion in the goblet cells of the lung epithelium.

Pulmonary eosinophils were quantified as described in a previous study [20]. Briefly, sections of lung were examined by a pathologist blinded from the study. Four random foci were selected and eosinophils were counted per viewing field (400x magnification) and averaged for each lung.

For evaluation of mucus secretion, we used a semi-quantitative scoring system on PAS-stained sections [21]. We graded the presence of mucus with the central and peripheral airway epithelium goblet cell mucus content using as follows: grade 0, no PAS staining; grade 1, 25% or less of the airway epithelium had PAS staining; grade 2, 26–50% of the airway epithelium had PAS staining; grade 3, 51–75% of the airway epithelium had PAS staining; and grade 4, >75% of the airway epithelium had PAS staining.

### Measurement of OVA-specific IgE, IgG antibodies

OVA-specific IgE, IgG1, IgG2a antibodies were measured by ELISA in the blood samples. Briefly, 96 well plates were coated with 5 ug OVA in a 100 uL of coating buffer overnight at 4°C. Nonspecific binding was blocked with 2% bovine serum albumin at 37°C for 1 hr. After incubation of the test sera for 2 hr, the plates were incubated with biotin-conjugated mouse IgE antibody (Southern Biotech, Birmingham, AL, USA), anti-IgG1 antibody, anti-IgG2a antibody at 37°C for 1 hr. After washing, 1∶1000 diluted streptavidin-HRP (BD Bioscience, San Jose, CA, USA) were added and plates were incubated at 37°C for 30 min. The reaction was developed with stabilized chromogen substrate (Biosource, Camarillo, CA, USA) at room temperature for 30 min and stopped by adding 2N H_2_SO_4_. The optical density was measured at 450 nm.

### Cytokine analysis using real time PCR

RNA was extracted from the homogenized lung tissue using Trizol (GIBCO BRL, Grand island, NY, USA) for the measurement of mRNA expressions of cytokines and alarmins. RNA (2 ug) was converted into cDNA using oligo (dT) and revere transcription enzyme (Promega, Madison, WI, USA). Expression of specific gene mRNA was measured using ABI 7500 real-time PCR system (Applied Biosystems, Foster, CA, USA) with cDNA, primer solution, and SYBR Green master mix (Applied Biosystems). Primer sequences of each gene used in the experiment were as follows: IL-4, forward 5′- ACTTGAGAGAGATCATCGGCA-3′ and reverse 5′- AGCTCCATGAGAACACTAGAGTT-3′; IL-5, forward 5′- AGCACAGTGGTGAAAGAGACCTT-3′ and reverse 5′- CATCGTCTCATTGCTTGTCAACA-3′; IL-13, forward 5′- CCTCTGACCCTTAAGGAGCTTAT-3′ and reverse 5′- CGTTGCACAGGGGAGTCT-3′; beta-actin, forward 5′- AGTGTGACGTTGACATCCGT-3′ and reverse 5′- GCAGCTCAGTAACAGTCCGC-3′.

### Cytokine analysis with multiplex ELISA

Interleukin-1β (IL-1β), IL-4, IL-5, IL-6, IL-10, IL-13, IL-17, tumor necrosis factor-α (TNF-α) and interferon-γ (IFN-γ) was measured using Bio-Plex cytokine assay (Bio-Plex Laboratories Inc., Hercules, CA, USA) in the BAL fluid as the manufacturer's instruction. Briefly, premixed magnetic beads coated (50 uL) with target capture antibodies were transferred to each well of the filtration plate and washed twice with Bio-Plex wash buffer. Premixed standards or diluted samples (50 uL) were added to each well containing washed beads. The plate was incubated for 30 min at room temperature with a shaker at low speed (300 rpm). After incubation and washing, premixed detection antibodies (25 uL) were added to each well. Then the plate was incubated for 30 min again. After incubation and washing, streptavidin-PE (50 uL) was added to each well. The incubation was terminated after shaking for 10 min at room temperature. After washing, the beads were re-suspended in 125 uL of Bio-Plex assay buffer. Beads were read on the Bio-Plex suspension array system, and the data were analyzed using Bio-Plex Manager software version 3.0.

### In vitro hypochlorite exposure

To evaluate *in vitro* immunological effect of low dose NaOCl exposure aggravating allergic inflammation, we measured thymic stromal lymphopoietin (TSLP), IL-33, and IL-1β expression after treating low dose NaOCl on the epithelial and macrophage cell lines.

AMJ2-C11 (mouse macrophage cells, ATCC; CRL-2456) was used for IL-1β, caspase-1, and IL-18 expression. Cells were treated with 0.0001∼0.005% in serum free media. Cell supernatants and lysates samples were used for the analysis of mRNA and protein expression. MLE12 (mouse lung epithelial cells, ATCC; CRL-2110) was used for IL-33 expression and treated with 0.001% NaOCl up to 96 hrs. A549 (human epithelial lung carcinoma cells, American Type Culture Collection (ATCC), MD, USA; CRL-185) was used for the evaluation of TSLP. Cells were treated with 0.001% and 0.003% NaOCl for 24 hr and 48 hr and cell lysates were used for western blot.

IL-1β, IL-33 and TSLP mRNA expressions from NaOCl-treated cells were measured using real-time PCR as previously described method. Primer sequence of each gene was as follows; IL-1β, forward 5′-GCAACTGTTCCTGAACTCAACT-3′ and reverse 5′-ATCTTTTGGGGTCCGTCAACT-3′; IL-33, forward 5′- TGAGAAACCTGAAAAATGAGACCTAGA-3′ and reverse 5′- CTGCGGTGCTGCTGAACTT-3′; TSLP, forward 5′-TATGAGTGGGACCAAAAGTACCG-3′ and reverse 5′-AGTAAGGCAATGTGGCCGATT-3′.

### Western blot

In order to detect the activated form of IL-1β, caspase-1 and IL-18 from NaOCl-treated AMJ2-C11 cells, Culture supernatants concentrated using Amicon Ultra Centrifugal Filters for Protein Purification and Concentration (Millipore, Billerica, MA, USA) and cell lysates were used. Sodium dodecyl sulfate (SDS) and β-mercaptoethanol were added to all samples, and final sample protein concentrations were adjusted by adding more lysis buffer. Proteins (20 µg/well) from culture supernatants and cell lysates were separated by 10% SDS-polyacrylamide gel electrophoresis and blotted onto PVDF membranes. The membranes were then probed with anti-mouse IL1β (Abcam, Cambridge, MA, USA), caspase-1 (Santa Cruz Biotech, Santa Cruz, CA, USA) and IL-18 (Santa Cruz Biotech) antibodies prior to incubation with HRP-conjugated secondary antibodies. The blots were developed using the Super-Signal West Dura chemoluminiscence system (Pierce, Perbio Science, Helsingborg, Sweden) according to the manufacturer's instructions. The bands representative were detected by FluorChem ™ HD2 Imager (Cell Biosciences). TSLP expression was analyzed with western blot in the cell lysates from NaOCl-treated A549 cells.

### Statistical analysis

Statistical differences among groups were assessed using Kruskal-Wallis test or Mann-Whtiney test. For multiple comparisons Kruskal-Wallis test was used initially, and if significant differences were found, Mann-Whtiney test was used for the comparison of statistical difference between two groups. Statistical analyses were performed using SPSS ver. 13.0 (SPSS, Inc., Chicago, IL, USA); a *p*-value less than 0.05 was deemed to indicate statistical significance.

## Results

### Effects of low dose chronic chlorine exposure on allergen-induced airway responsiveness and inflammation

**Figure 2:**
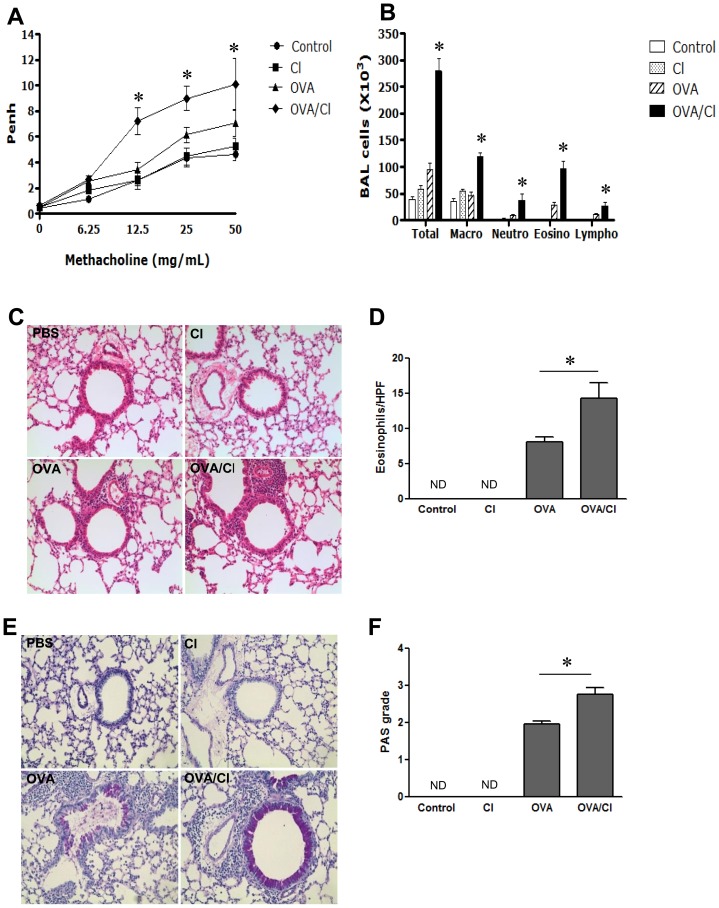
Effect of low dose chronic chlorine exposure on allergen-induced airway hyperresponsiveness and inflammation 6 weeks old BALB/c mice were sensitized and challenged with OVA allergen with or without low dose chlorine exposure. A, Airway hyperresponsiveness was determined by whole body phlethysmography *p<0.05 vs. other groups. B, Lung inflammatory response were measured by BAL cell recovery. Macro, macrophages; Neutro, neutrophils; Eosino, eosinophils; Lympho, lymphocytes. *p<0.05 vs. other groups. C, H&E staining (x200). D, Quantification of eosinophil in the lung tissue. E, PAS staining (x200), F: Semi-quantified mucus index score in the PAS staining. Values in panels A, B, D and F are mean ± SEM of evaluations in a minimum of 5 mice. Panel C and E is a representative of a minimum of four similar experiments.

To see the effect of chlorine in allergen-induced airway responsiveness and inflammation, BALB/c mice were sensitized and challenged with OVA in the presence and absence of chlorine exposure. Our studies demonstrated that chronic exposure to low dose chlorine did not induce AHR when it was exposed alone without allergen exposure. However, OVA-sensitized and challenged mice exposed to low dose chlorine chronically (OVA/Cl) showed significantly higher AHR than the mice only with OVA-sensitization and challenge without chlorine exposure (OVA) (Fig. 2A). In BAL fluid analysis, the mice with chronic exposure of low dose chlorine showed increased recovery of macrophages than the mice without any challenge (controls), to the comparable levels with OVA-challenged ones. However, the OVA-challenged mice with low dose chlorine exposure demonstrated significantly increased BAL recovery of eosinophils, neutrophils, and lymphocytes as well as macrophages compared to the mice sensitized and challenged only with OVA (Fig. 2B). In the histological examination, no significant inflammation was noted in the lungs of controls and the mice exposed to chlorine alone (Fig. 2C). However, as was in the BAL inflammation, the mice challenged with OVA alone or together with chlorine exposure showed significant infiltration of inflammatory cells in the lung. Of these, the group of mice with OVA/Cl showed more prominent increases in the influx of inflammatory cells into peribronchial and perivascular regions of the lung than the mice only with OVA sensitization and challenge (Fig. 2C). Eosinophil was significantly increased in the lung tissue of OVA/Cl group of mice compared to that of OVA group (Fig. 2D). In addition, significantly higher mucus index score also was noted in OVA/Cl group than in the OVA group in the PAS staining (Fig. 2E–F).

### Effects of low dose chronic chlorine exposure on allergen sensitization

As a first step to understand the mechanism of chlorine regulation of allergen-induced inflammation and AHR, serum OVA-specific IgE and IgG levels also measured to evaluate whether the chronic exposure to low dose chlorine affect allergen sensitization. No significant difference were found in the production of OVA-specific IgE, IgG1, and IgG2a levels in the OVA/Cl group compared with OVA group (Fig. S1), suggesting that chlorine exposure did not affect OVA allergen sensitization process.

### Effects of low dose chronic chlorine exposure on allergen-stimulated Th2 cytokine expression

**Figure 3:**
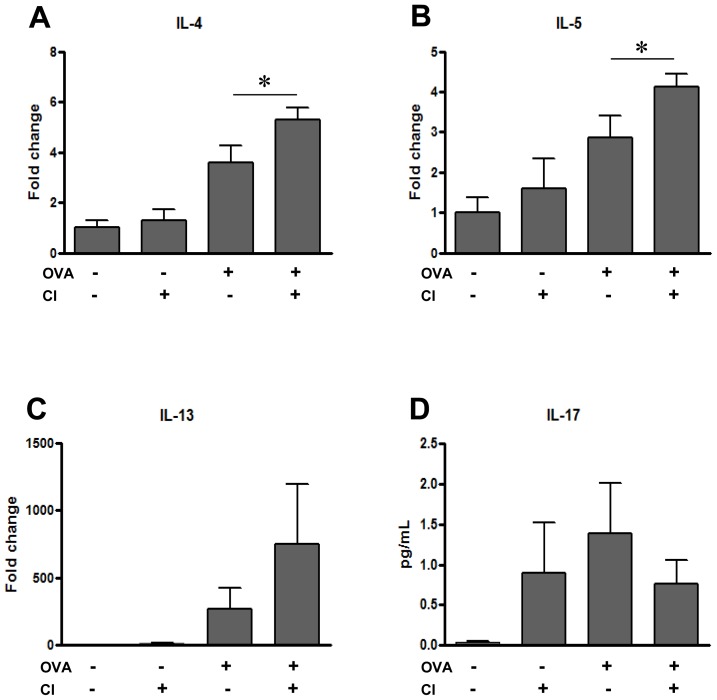
Effect of low dose chronic chlorine exposure on Th2 cytokine and IL-17 expression in OVA sensitized and challenged mice. A-C, the mRNA expression of IL-4 (A), IL-5(B) and IL-13(C) was evaluated by RT-qPCR. D. The level of IL-17 in BAL was measured by ELISA. Values in these panels are mean ± SEM of evaluations in a minimum of 5 mice. *p<0.05 vs. OVA only group.

To understand the mechanism that low dose chlorine aggravates AHR and airway inflammation in OVA asthma mouse model, first we evaluate the expression of Th1 and Th2 cytokines in these mice. The real-time qPCR analysis revealed a significant increase in the expression of Th2 cytokines, such as IL-4 and IL-5 in OVA/Cl group of mice compared with OVA group. IL-13 was increased in OVA/Cl group, but did not reach statistical significance compared with OVA group (Fig. 3A–C). Level of IL-5 in BAL fluid was also significantly higher in OVA/Cl group than OVA group (Fig. S2B). Interestingly, IL-17 level was increased in the mice with chlorine only exposure compared to controls, but no significant differences were noted between the groups of mice OVA only or OVA/Cl (Fig. 3D). In contrast, the levels of Th1 cytokines, such as IFN-γ or TNF-a, were neither induced nor modulated with chlorine exposure (data not shown). These studies demonstrated that chronic exposure of chlorine augments allergen-stimulated expression of Th2 cytokines that may underlie the exaggerated allergic inflammation and physiological response in the lung.

### Effect of low dose chronic chlorine exposure on the inflammasome activation

**Figure 4:**
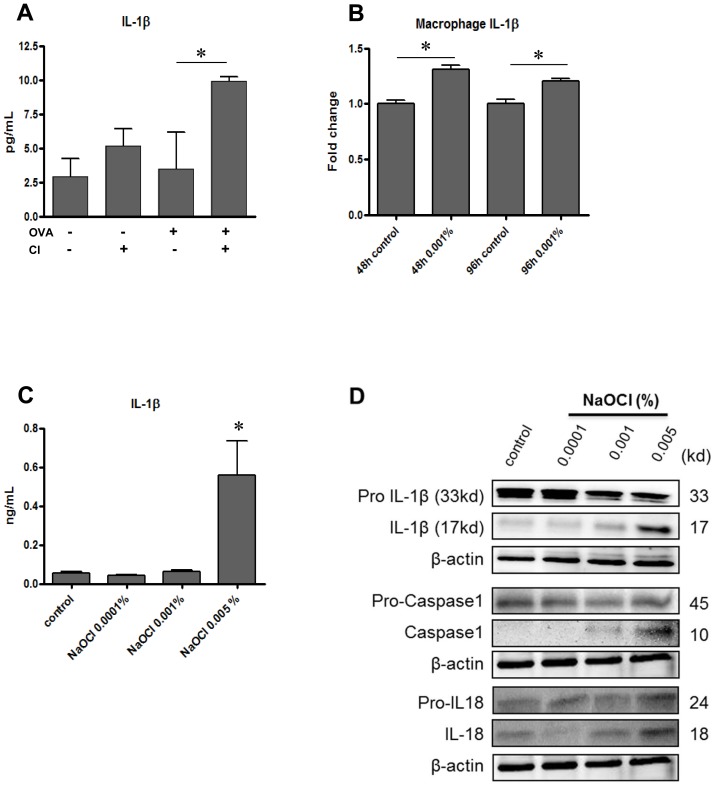
Effect of low dose chronic chlorine exposure on the molecules associated with inflammasome activation. A, IL-1β levels in the BAL from OVA-sensitized and challenged mice with or without chlorine exposure were measured by ELISA. *p<0.05 vs. OVA only group. B, The mRNA expressions of IL-1β were measured by RT-qPCR after 48 and 96 hrs stimulation with 0.001% NaOCl. *p<0.05 vs. control. C, IL-1β levels were measured using ELISA after low dose NaOCl treatments in AMJ2-C11 mouse macrophage cells. *p<0.05 vs. control. D, Western blot evaluation on the molecules associated with inflammasome activation in the macrophages with and without exposure of NaOCl (IL-1β and caspase-1 in the cell supernatant, and IL-18 in the cell lysates). Values in panels in A, B and C are mean ± SEM. Panel D is a representative of a minimum of three similar experiments.

Next studies were undertaken to see if chlorine exposure modulate the expression of mediators potentially associated with inflammasome activation pathway. Interestingly, significant increase in IL-1β level was found in BAL fluid of the OVA/Cl group compared to controls or OVA group (Fig. 4A). In support of this *in vivo* finding, we further defined direct chlorine regulation of IL-1β expression using AMJ2-C11 murine macrophage cells after stimulation of low dose chlorine. As shown in Fig. 4B and 4C, the mRNA and protein expression of IL-1β were significantly increased in the macrophages treated with chlorine up to 96 hrs. These studies strongly suggest that chlorine is a powerful stimulant of IL-1β *in vivo* and *in vitro*. To further define contribution of chlorine to the inflammasome activation, we evaluated the cleavage of IL-1β, caspase-1, and IL-18 using Western blot (Fig. 4D). These analyses demonstrated that cleaved active forms of IL-1β (p17), caspase-1 (p10), and IL-18 (p18) were all significantly increased in the macrophage exposed to low dose chlorine (0.001% to 0.005% of sodium hypochlorite) compared to vehicle-treated ones. These data indicates significant inflammasome activation with chlorine stimulation in the macrophage.

### Effects of low dose chronic chlorine exposure on the expression of IL-33 and TSLP

**Figure 5:**
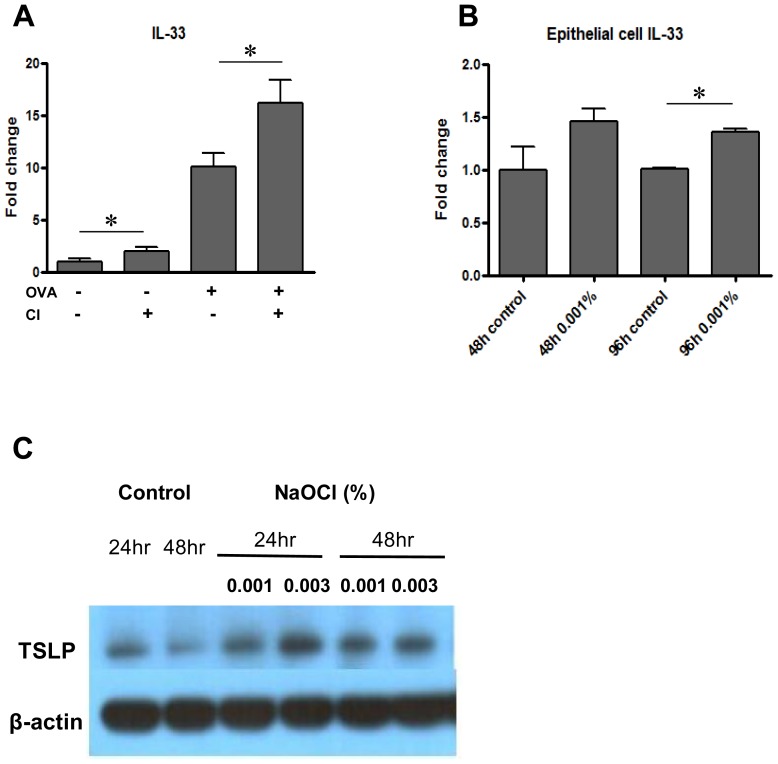
Effect of low dose chronic chlorine exposure on the expression of epithelial alarmines. A, The levels of IL-33 expression in the lungs from the mice sensitized and challenged by OVA with or without chlorine exposure were evaluated by RT-qPCR. *p<0.05 vs. groups without chlorine exposure. B, The mRNA expressions of IL-33 in murine MLE12 epithelial cells were measured by qRT-PCR after 48 and 96 hrs stimulation of 0.001% NaOCl. *p<0.05 vs. control. C, TSLP protein expression was evaluated by Western blot analysis. Values in panels A and B are mean ± SEM of evaluations in a minimum of 5 mice. Panel C is a representative of a minimum of three similar experiments.

It has been shown that epithelial cell damage caused by a number of chemicals or viral exposure significantly contributed to the increased allergic responses and asthma pathogenesis through the expression of alarmins such as IL-33 and TSLP [22]. IL-33, but not TSLP, is mostly dispensable for antigen-specific Th2 cell differentiation and antigen-specific IgE production [23]. Since our studies showed no significant changes at the levels of allergen (OVA)-specific IgE with chlorine exposure, studies were first undertaken to define the chlorine regulation of IL-33. As shown in Fig. 5A, the mice exposed to chlorine showed increased expression of IL-33 compared to controls. In addition, the mice challenged with OVA together with chlorine significantly increased the expression of IL-33 compared to the group of mice with OVA only. To see direct effect of chlorine on the induction of IL-33, we stimulated the MLE12 murine epithelial cells with low dose chlorine *in vitro* and evaluated the level of mRNA expression of IL-33 by real-time PCR. The addition of chlorine consistently stimulates the expression of IL-33 up to 96 hours compared with controls (Fig. 5B). These studies suggest that chorine stimulate the expression of IL-33, an alarmin that has profound ability to enhance allergic inflammation [24]. On the other hand, the chronic chlorine exposure did not modulate the expression of TSLP expression in the lung *in vivo* (Fig. S3). However, increased expression of TSLP was noted after stimulation with low dose chlorine in A549 human epithelial cells (Fig. 5C).

## Discussion

Acute exposure to high dose chlorine was experimentally demonstrated to induce AHR, airway inflammation, and acute pulmonary edema [16]–[18]. However, study to demonstrate the role of chronic exposure to low dose chlorine in the development or aggravation of asthma has been rarely explored yet either in clinical studies or experimental investigations. In this study, AHR and airway inflammation were aggravated in murine asthmatic airways while low dose chlorine exposure alone did not influence on the development of AHR and airway inflammation. Using this model, we investigated the underlying immune mechanism of asthma aggravation induced by chronic exposure to low dose chlorine as like occupational exposure of cleaner and house wife, and chlorine products users.

Chlorine is an irritating gas and can be a highly reactive oxidant forming to hydrogen chloride (HCl) or hypochlorous acid (HOCl) after reacting with water in human airway mucosa [25]. Much attention has not been paid on low dose chlorine exposure because it does not generally evoke any acute symptoms. However, there are several epidemiological and clinical reports regarding this issue. In European countries, the prevalence of asthma and allergic disease was significantly higher in children swimming in chlorinated pool [26]–[28]. Especially it was reported that children with high serum IgE had maximum 9 fold of asthma risk even without family history of asthma when they swim in the chlorinate pool for a long time [26]. In adults, competitive swimmers who spend more time in the pool than recreational swimmers showed increased level of surfactant protein (SP-A, SP-B) or Clara cell protein (CC16), which suggest longer exposure to swimming pool increase epithelial damage and exposure to chlorine compound might be related to this finding [29]. Chlorine was noticed as a cause of occupational asthma in swimming athletics, recreational swimming, and indoor swimming pool workers [6]–[8], [30]. Inhaled chlorine gas derived from chlorine compound in pool can be converted to ROS and affect bronchial mucosa [25]. Highly volatile trichloramine or aerosol of monochloramine, dichloramine, and hypochlorous acid formed in the pool might give damage on airway epithelium [2]. Chlorine gas can also be exposed in daily life. Bleaching solution, sodium hypochlorite can produce large amount of chlorine gas when it reacts to acid, and the production of chlorine gas is easily recognizable because it is visible and highly irritating in this case. On the other hand, sodium hypochlorite can produce small amount of naturally vaporized chlorine gas, which usually do not irritate airway mucosa [31]. However, cleaners who are occupationally exposed to chlorine gas for a long time are noted to have increased prevalence of asthma [9], [32]. Thus, even low level of chlorine may affect airway mucosa when exposed chronically.

Our studies clearly demonstrate that low dose chlorine exposure significantly aggravate allergen (OVA)-induced inflammation and AHR. The chlorine exposure enhances Th2, but not Th1, inflammation in the lung with increased expression of IL-4 and IL-5 expression. Interestingly, IL-17 expression in the lung was also induced by low dose chlorine exposure, but was not synergistically increased in the group mice with OVA/Cl compared to the mice with OVA only. These studies suggest that chlorine exposure significantly affect the development and progression of asthmatic airways by modulating cytokine milieu to drive Th2 inflammation. However, although chlorine exposure itself increases the expression of IL-17 in the lung, the biological role of IL-17 in this pathology remains to be determined.

To define the effects of low dose chlorine exposure in asthma aggravation, we first evaluated the expression of cytokines and proinflammatory mediators in BAL and lung tissue of the allergic animal model induced by co-exposure of low dose chlorine and OVA. Interestingly, we noted a significant increase in the expression of IL-1β along with Th2 cytokines IL-4 and IL-5, *in vivo* in the lung and *in vitro* after exposure of chlorine. IL-1β is largely expressed in macrophage or monocytes through the activation of TLR signaling or inflammasome induced by various endogenous and exogenous substance [33]–[35]. Although ROS was not measured in this study, reactive chlorine species have similar characteristics with ROS and chlorine exposure is known to be associated with ROS generation [25], [36]. ROS and epithelial damage caused by oxidative stress of chlorine may produce alarmins and cytokines, that activate TLR or NLRP3 inflammasome, and promote the production of IL-1β [33]. Thus, we can easily envision that the increased expression IL-1β in the BAL fluid after chlorine stimulation suggest that inflammasome activation pathways could be implicated in the biological effect of chlorine. In support of this notion, our *in vitro* studies further demonstrated that increase in the activated form of caspase-1, IL-1β, and IL-18 (cleavage from pro-form) after chlorine exposure. Although this is the first studies demonstrate that the chlorine exposure activates the inflammasome pathways, the exact biological role of inflammasome activated caspase-1, IL-1β, and IL-18 in allergic inflammation and AHR warrants further investigation.

Chlorine gas is known to induce epithelial damage via production of reactive oxidants [2], [5], [25]. However, little is understood how this epithelial damage contribute to the development of aggravation of allergic reaction. In our study, low dose chlorine induces neither AHR nor airway inflammation by itself, but it can augment AHR and airway inflammation induced by OVA. This finding suggests chlorine may act as an enhancer of immune reaction in the development of allergen-induced immune reaction. Alarmin, endogenous molecule derived from damaged airway epithelium may be involved in this mechanism by producing danger signals [35], [37]. IL-33, a member of IL-1 family cytokine, is secreted from the damaged epithelial cell and can be also classified as one of alarmins [35], [38]. IL-33 can interact to ST receptors also known as IL-RL1 largely located on the Th2 helper cell and mast cell and have been understood to play a crucial role in the development of allergic disease such as asthma and atopic dermatitis [38]. In addition, TSLP derived from epithelial cell is well recognized to play an important role in the early phase of allergic inflammation [39]. TSLP promote the environment for Th2 inflammation by inducing dendritic cell activation and maturation, increasing MHC expression, and producing macrophage-derived chemokine (MDC) and thymus and activation-regulated chemokine (TARC) [40], [41]. In the present study, IL-33 significantly increased in the mice which were exposed to the low dose chlorine with OVA. Furthermore, IL-33 expression was enhanced by low dose hypochlorite in the *in vitro* experiment using mouse epithelial cell. Although the enhanced expression of TSLP was not found in the animal experiment, significant increase of TSLP was observed in the human and mouse epithelial cell after low dose chlorine exposure. It suggests that TSLP also may have a potential to contribute to the enhanced allergic responses by chlorine exposure together with IL-33. The specific role and contribution of IL-33 or TSLP in the effects of chlorine in pathogenesis of allergic inflammation and physiologic response need to be further defined.

Chlorine has been supposed to contribute to the sensitization of allergen by destructing epithelial junction barrier and increasing allergen permeability [2]. Our study had a limitation to evaluate the effect of chlorine on allergen sensitization, the first step of asthma development, because OVA sensitization was induced via intraperitoneal route. However, recent study by Hox et al. also demonstrated that allergen-specific sensitization was not affect by repetitive exposure to low dose chlorine although it increased AHR [42]. In our study, increased expression of IL-4 and IL-5 represent the enhancement of Th2 inflammation, but these finding might be caused by innate immune response related to the action of alarmins such as IL-33 and TSLP rather than adaptive immune response. IL-33 can induce Th2 inflammation by activating mast cell, macrophage, and innate lymphoid cells regardless of adaptive immune response [24], [43]. TSLP can activate innate immune cells such as mast cell, basophil, and invariant natural kill T cell to secret Th2 cytokines [44], [45]. Since IL-33, but not TSLP, is mostly dispensable for antigen-specific IgE production and our studies showed no significance difference in the OVA allergen-specific IgE levels with and without chlorine exposure, we speculate that IL-33 is more relevant to the biological effect of chlorine than TSLP. It is also intriguing to speculate that chlorine-stimulated epithelial alarmins could play a major role in the inflammasome activation that lead to the secretion and activation of proinflammtory cytokine IL-1β. If this is the case, further investigation on the specific role of IL-33 or TSLP in these activation pathways could be important to understand the mechanism of chlorine effect on allergic inflammation and physiologic responses.

In conclusion, our studies demonstrated that low dose repeated chronic exposure of chlorine gas vaporized from sodium hypochlorite aggravates allergen-induce inflammation and AHR. These studies also identified that chlorine exposure significantly increases the expression of epithelial alarmins such as IL-33 or TSLP together with IL-1β *in vivo* and *in vitro*. These findings led us to speculate that alarmin-inflammasome activation pathways might be implicated as a mechanism of biologic effect of chlorine in the pathogenesis of asthmatic airways.

## Supporting Information

Figure S1 **Effect of low dose chronic chlorine exposure on allergen sensitization.** The levels of OVA-specific IgE or IgG1, IgG2a in the serum were measured at the time of sacrifice using ELISA. Values in these panels are mean ± SEM of evaluations in a minimum of 5 mice. NS, no statistical significance compared to OVA only mice.
(TIF)

Figure S2 **The levels of Th2 cytokines in the BAL.** IL-4 (A) and IL-5 (B) measured using ELISA. Values in these panels are mean ± SEM of evaluations in a minimum of 5 mice. *p<0.05 vs. OVA only group.
(TIF)

Figure S3 **TSLP mRNA expression in the lung.** mRNA expression of TSLP measured using RT-qPCR. Values in these panels are mean ± SEM of evaluations in a minimum of 5 mice.
(TIF)
