# Supplementary figures and images for: Chronic Low Dose Chlorine Exposure Aggravates Allergic Inflammation and Airway Hyperresponsiveness and Activates Inflammasome Pathway
Source: PLoS One. 2014 Sep 9;9(9):e106861. doi: 10.1371/journal.pone.0106861 (PMC4159271; doi:10.1371/journal.pone.0106861)

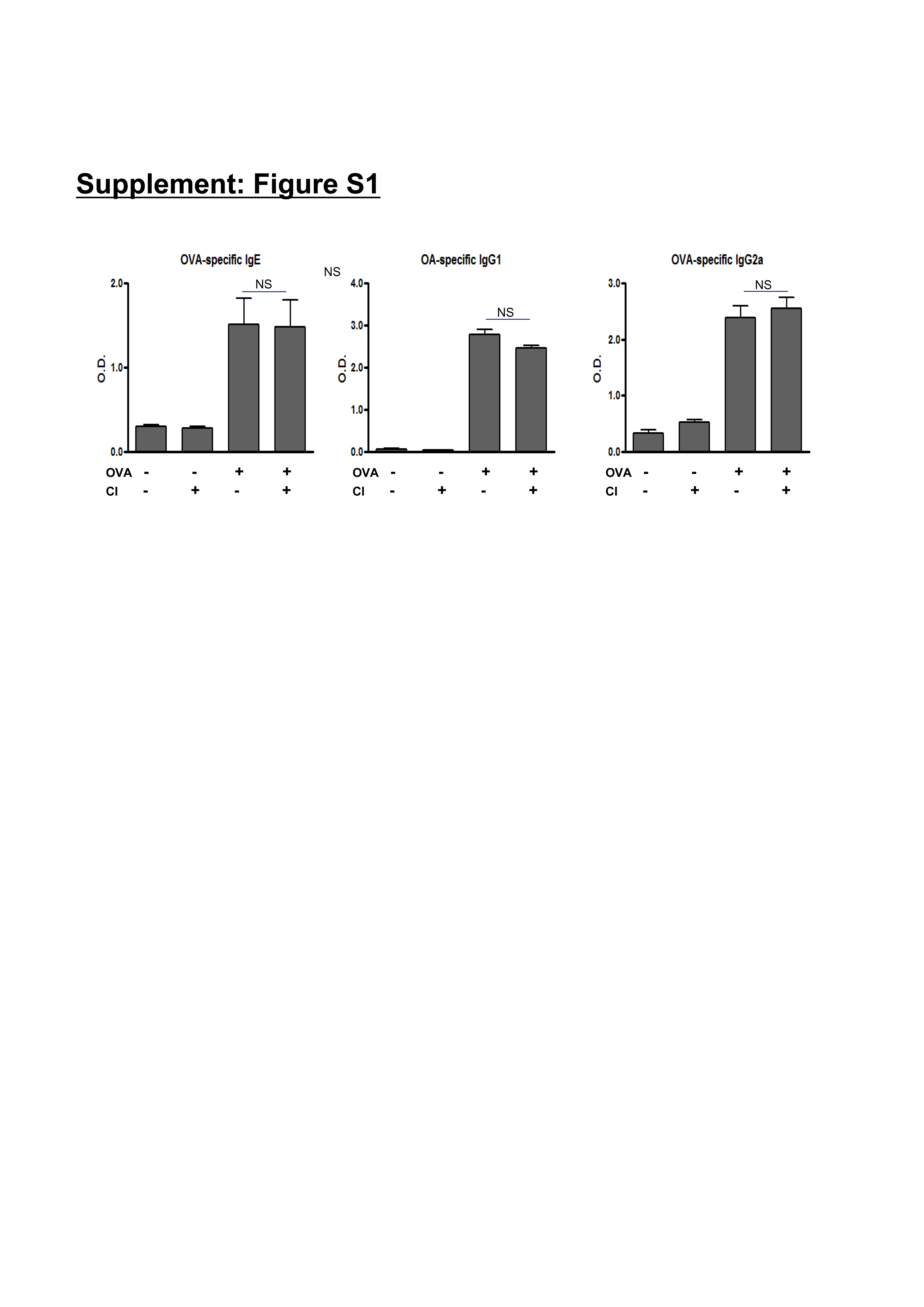

Supplement: Figure S1 — Effect of low dose chronic chlorine exposure on allergen sensitization. The levels of OVA-specific IgE or IgG1, IgG2a in the serum were measured at the time of sacrifice using ELISA. Values in these panels are mean ± SEM of evaluations in a minimum of 5 mice. NS, no statistical significance compared to OVA only mice. (TIF) [file pone.0106861.s001.tif]

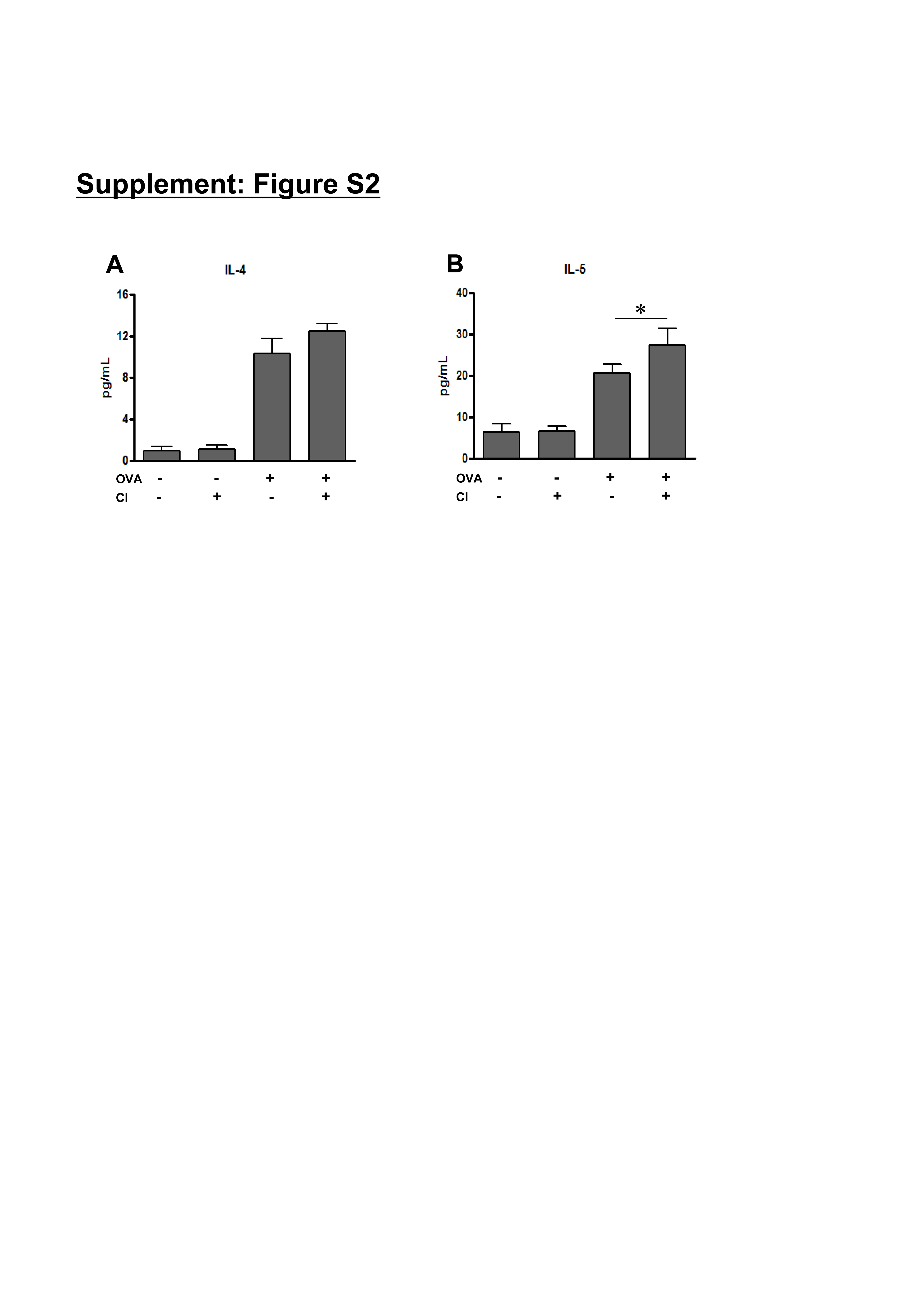

Supplement: Figure S2 — The levels of Th2 cytokines in the BAL. IL-4 (A) and IL-5 (B) measured using ELISA. Values in these panels are mean ± SEM of evaluations in a minimum of 5 mice. *p<0.05 vs. OVA only group. (TIF) [file pone.0106861.s002.tif]

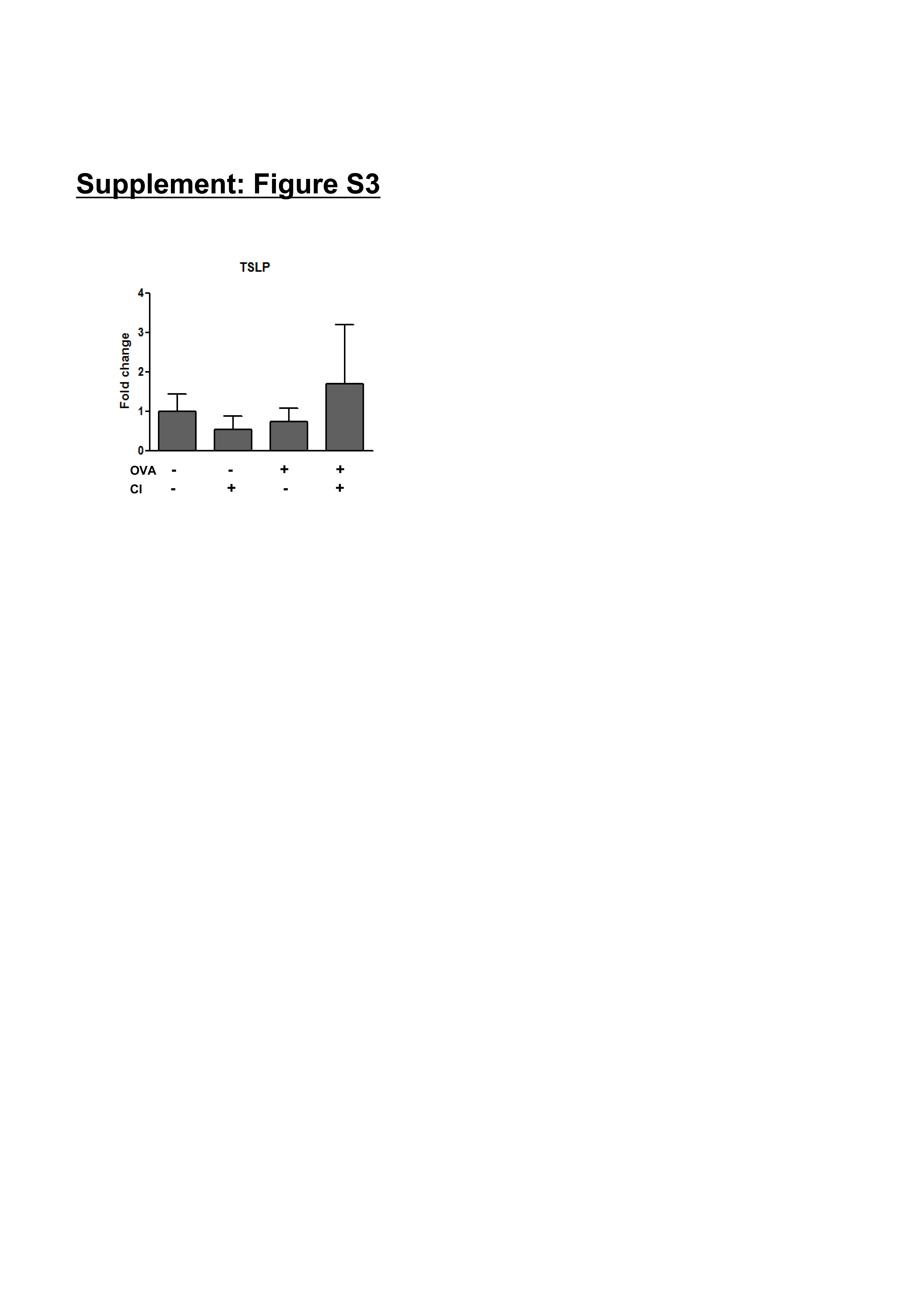

Supplement: Figure S3 — TSLP mRNA expression in the lung. mRNA expression of TSLP measured using RT-qPCR. Values in these panels are mean ± SEM of evaluations in a minimum of 5 mice. (TIF) [file pone.0106861.s003.tif]
